# A Research Protocol to Test the Effectiveness of Text Messaging and Reminder Calls to Increase Service Use Referrals in a Community Engagement Program

**DOI:** 10.2196/resprot.5854

**Published:** 2016-06-28

**Authors:** Deepthi Satheesa Varma, Mark Hart, Denise Sonya McIntyre, Evan Kwiatkowski, Linda Bauer Cottler

**Affiliations:** ^1^ College of Public Health and Health Professions and College of Medicine Department of Epidemiology University of Florida Gainesville, FL United States

**Keywords:** text messaging, reminder calls, community engagement, referral use, mHealth

## Abstract

**Background:**

Mobile phoned–based interventions have been increasingly used in clinical populations to improve health and health care delivery. The literature has shown that mobile phone–based text messages (short message service, SMS) are instantaneous, cost effective, and have less chance of being misplaced. Studies using mobile phone based–text messages have reported text messages as effective reminders that have resulted in increased appointment attendance, adherence to treatment, and better self-management. There have been no reports of adverse events when using text messaging in terms of misreading or misinterpreting data, transmitting inaccurate data, losing verbal or nonverbal communication cues, privacy issues, or failure or delay in message delivery. However, the literature has cited a need for personalized messages that are more responsive to individual needs. In addition, there has been a dearth of information on the use of reminders in nonclinical populations.

**Objective:**

The goal of this study is to assess the effectiveness of adding reminders in the form of text messaging versus reminder calls versus text messages and reminder calls to increase use of service referrals provided through community outreach.

**Methods:**

A total of 300 participants will be recruited for the study. Each participant will be randomized to one of three arms: a group that receives only reminder calls (CALLSONLY); a group that receives only text message reminders (TEXTONLY); and a group that receives both reminder calls and text messages (CALLS+TEXT). All groups will receive their reminder intervention on the 15th and 45th day after baseline when they receive medical and social service referrals from the community health workers (CHWs). A standard script will be used to administer the call and text reminders and a 15-item telephone-based satisfaction survey will be administered to assess the participant satisfaction with the process of receiving periodic reminders.

**Results:**

The study is in the recruitment and follow-up phase. The authors anticipate completion of recruitment, interventions, and data entry by July 2016. Preliminary results are expected to be available by September 2016.

**Conclusions:**

This study will provide an opportunity to test the effectiveness of mobile-based interventions on nonclinical, community-recruited populations. In particular, such a protocol would increase the effectiveness of a community-based engagement program by instating a formal reminder system for all program members who receive social and/or medical service referrals during outreach in the community. Findings from this study would guide the development and implementation of reminder protocols for community-based engagement programs nationwide.

## Introduction

### Background

There has been a significant increase in the literature on mobile phone–based interventions (mHealth) to improve health care among clinical populations. At the end of 2014, over 3.6 billion people had at least one mobile subscription. It is estimated that by 2020, 4.6 billion people will have a mobile subscription [1]. Recent studies have expressed the need for harnessing the increasing availability and acceptability of mobile phones in health care to enhance contact with health services, self-management, and broadening the scope of mHealth applications [2,3]. Mobile phone–based text messages (short message service, SMS) have been described as ‘instantaneous’, ‘direct’, ‘mobile’, and ‘not invasive and ubiquitous’. They also have less chance of being misplaced [4,5]. Other benefits of text messages include the ability to deliver the message wherever the person is, reducing dependence on health care professionals [6,7]. The interventions are also reported to be cost effective and more economical than telephone or postal reminders [6-8]. Studies have shown text messages as effective health care appointment reminders that can result in increased attendance, adherence to treatment, self-management, and maintenance of chronic conditions such as cardiovascular health, asthma, diabetes, obesity, and hypertension [5,6,9-12]. Research on missed health care appointments with a clinical population has shown that mobile phone–based interventions such as periodic phone call reminders and text messages increase the rate of attendance [13].

A study on improvement in attendance in primary care found the text messaging reminder group attended the clinic at a higher rate compared with the control group (odds ratio (OR) 1.59, 95% confidence interval (CI) 1.17-2.17, *P*=.005) [8]. Another study among persons with chronic disease showed the nonattendance rates in the text messaging (OR=0.62, 95%CI 0.41-0.93, *P*=.020) and telephone reminder groups (OR=0.53, 95%CI 0.35-0.81, *P*=.003) were significantly lower than the control group [14]. Text messaging, in weekly intervals, was found to be an effective strategy to enhance adherence to antiretroviral therapy compared with standard care among human immunodeficiency virus (HIV) patients and in improving HIV viral load suppression [15]. Text messaging interventions for smoking cessation and for effective health care service delivery processes were also found to be effective [16,17]. A study on treatment compliance among obstructive sleep apnea patients showed that message reminders via a mHealth application not only resulted in significantly higher treatment compliance but also was satisfactory to patients [18]. A block-randomized control study using a website and SMS-based reminder system among adolescents with asthma has found improvements in self-reported medication adherence (*P*=.011), quality of life (*P*=.037), and self-efficacy (*P*=.016) [19].

There have been no reports of adverse events when using text messaging in terms of misreading or misinterpreting data, transmitting inaccurate data, losing verbal or nonverbal communication cues, privacy issues, or failure or delay in message delivery [6]. Qualitative studies on content and type of text messages highlight the need for tailored and personalized messages that are “responsive to individual needs” [10,20,21]. The literature has captured the use of mHealth applications and reminders systems among clinical populations; however, there has been a dearth of information on the use of reminders in nonclinical populations.

Based on the literature, we proposed and were funded by the University of Florida’s Clinical and Translation Science Award Program to carry out a pilot study aimed to apply the qualities of mobile phone–based interventions to improve the utilization rate of medical and social service referrals provided to community members by the community health workers (CHWs) at HealthStreet–the community engagement initiative at the University of Florida. HealthStreet aims to bridge the gap between the community and research by providing medical and social service referrals based on their individual needs and concerns while providing opportunities for community members to become involved in research. CHWs from HealthStreet assess community members at laundromats, grocery stores, libraries, hair salons, bus stops, senior centers, community centers, health fairs, and other places. The assessment includes demographic characteristics, medical history, health and neighborhood concerns, access to care, attitudes toward research, history of research participation, drug and alcohol history, and current medication use. Community members are then followed-up with at 30 and 60 days to assess use of services. Approximately 63% of HealthStreet’s participants are African American, 30% did not see a doctor in the last 6 months in spite of having at least one health concern, and approximately 41% of HealthStreet’s participants report having no medical insurance. Therefore, the medical and social service referrals provided by the CHWs are especially important to this population. Prior data on use of service referrals at HealthStreet showed that 81% of participants who received at least one referral did not use any relevant referrals. Nearly 1/3 of participants indicated a barrier to service use that would be resolved with a simple reminder. Several of them mentioned that they would have used the services, if they had had the referral details available when needed.

Using Fogg’s Behavior Model [22], based on the persuasive power of technology for behavior changes [23], we hypothesize that simple mobile phone–based reminders will act as a “motivator” and a “trigger,” thereby encouraging the individual to use the provided referral service. Further, by sending the details of the referrals during the reminder text or the call will also solve the barrier of losing the referral slip and/or not having the referral details at hand when required.

HealthStreet data also showed 72% of HealthStreet participants regularly use text messaging, which made mobile phone–based interventions an ideal choice for a pilot study. With the decreasing cost of mobile phones and text messaging services and increasing availability of different service providers, mobile phone–based interventions could be considered an economical and efficient way to communicate.

We proposed to develop and test the effectiveness of a mobile phone–based intervention–referral reminder calls (CALLSONLY) versus text message reminders (TEXTONLY) versus both text message and reminder calls (CALLS+TEXT)– to increase the rate of service referral usage among HealthStreet participants. Additionally, this pilot study provided an opportunity to assess the HealthStreet participant’s satisfaction receiving reminder text messages.

### Overall Aim

The aim of this study is to increase use of service referrals provided through community outreach using a mHealth intervention (adding reminders in the form of text messages or reminder calls or text messages and reminder calls).

### Specific Objectives

The specific objectives for this study are to: (1) develop an effective and personalized text messaging system to increase use of referrals given by HealthStreet CHWs, (2) compare the effectiveness of three new interventions on referral use (TEXTONLY vs CALLSONLY vs CALLS+TEXT) at 30 and 60 days post baseline, and (3) deconstruct the intervention through a satisfaction/feasibility survey to understand HealthStreet participants’ satisfaction with these periodic reminders.

## Methods

### Study Design

**Figure 1:**
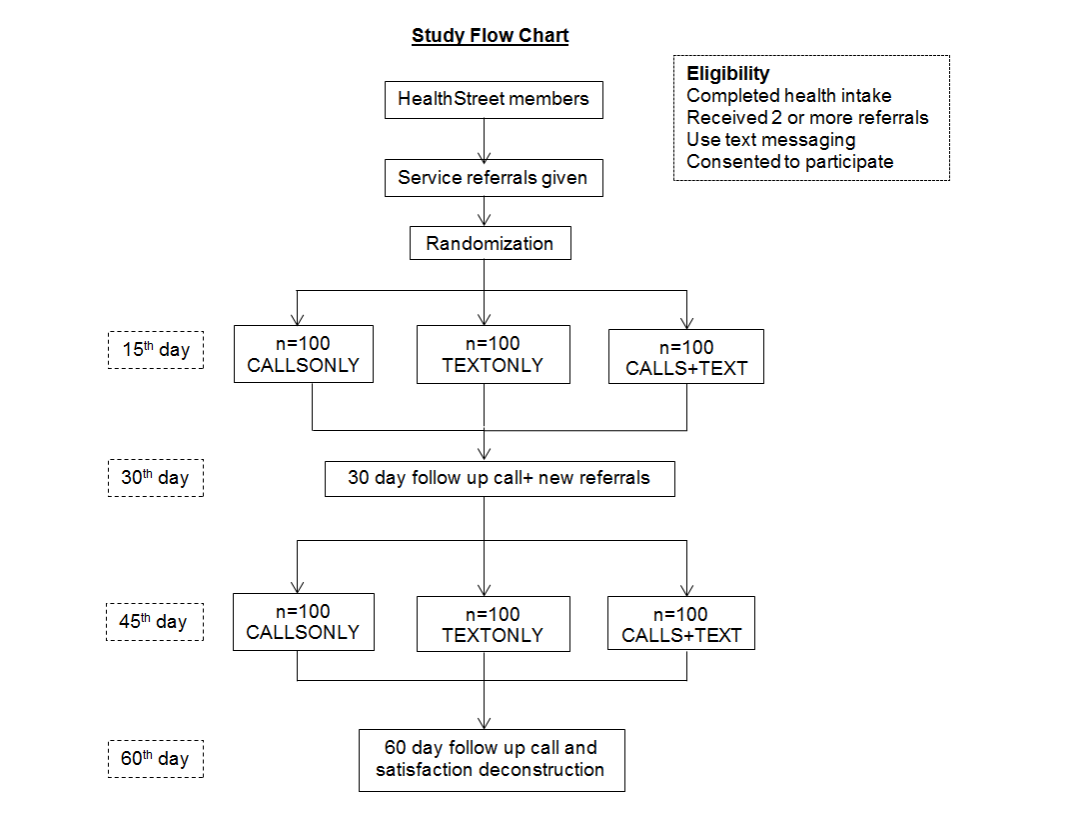
Study flow chart: recruitment and intervention groups.

A 12-month study is being conducted with 300 eligible community members from Alachua and Duval counties in North Central Florida. The study has three arms: a group that receives only reminder calls (CALLSONLY); a group that receives only text message reminders (TEXTONLY); and a group that receives both reminder calls and text messages (CALLS+TEXT) (Figure 1). All groups receive their reminder intervention on the 15^th^and 45^th^ day after baseline when they receive referrals from the CHWs. All groups then receive HealthStreet’s usual 30 and 60 day follow-up call that assesses use of the referrals provided at baseline. The 15^th^and 45^th^day, which falls in the middle of 0 to 30 days and 31 to 60 days, was decided for reminders because that will provide adequate number of days to use the referral from the baseline assessment date and/or the previous follow-up call date. All follow-up calls are made using the existing institutional review board–approved HealthStreet referral tracking protocol. Additionally, a satisfaction survey is also administered to all participants at 60 days to assess satisfaction with the different types of reminders and follow-up processes.

### Measures

To develop and implement a reminder call and text messaging protocol intervention to improve use of medical and social referrals provided to HealthStreet members by CHWs. The following measures are used:

Reminder calls: a standard script (Multimedia Appendix 1) is administered to remind the participant about the location, the date of contact with the HealthStreet CHWs as well as the service referrals provided to them at baseline. The call specifically reminds the person to use the referrals that were provided to them. Data on the use of referrals are collected only during the 30- and 60-day follow-up calls.

**Figure 2:**
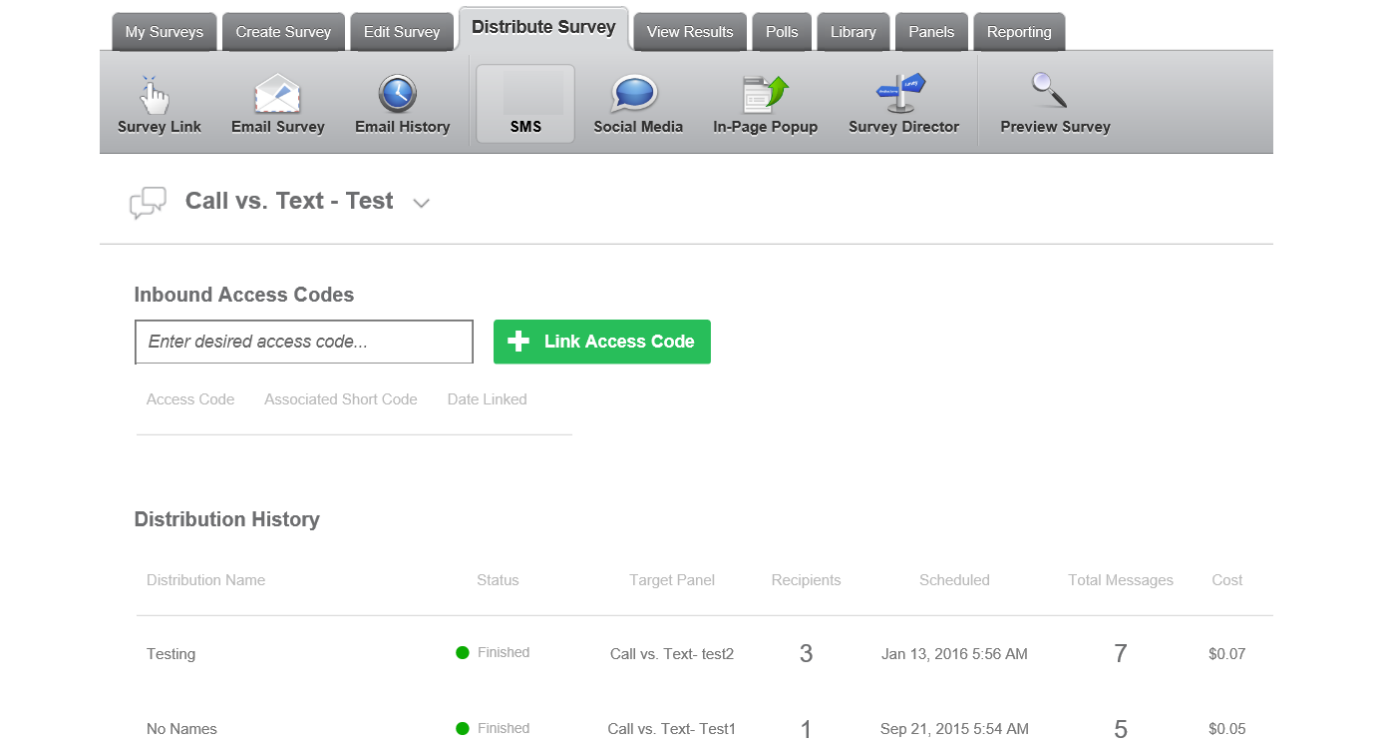
Qualtrics SMS surveys.

Text messaging: text messaging is conducted using the Qualtrics SMS (Figure 2) service, which is widely used in higher education research. The Qualtrics SMS service is compliant with the Health Insurance Portability and Accountability Act for data security and uses a secure local server for the data as it pushes out messages through an encrypted server. All data are stored in an internal secure server and only phone numbers and text messages are housed in the secure Qualtrics server. Once messages have been approved, and scheduled, they are sent out to their intended audience through an encrypted server that Qualtrics itself has the inability to track.  Ultimately, this tool is a one-way directional message service, with only a confirmation text from the participants coming back to the researchers.  This information, coupled with the backend analytic tools of Qualtrics, allows for data to be collected on who received the messages, which can then be compared with participant reported use of referrals.

**Figure 3:**
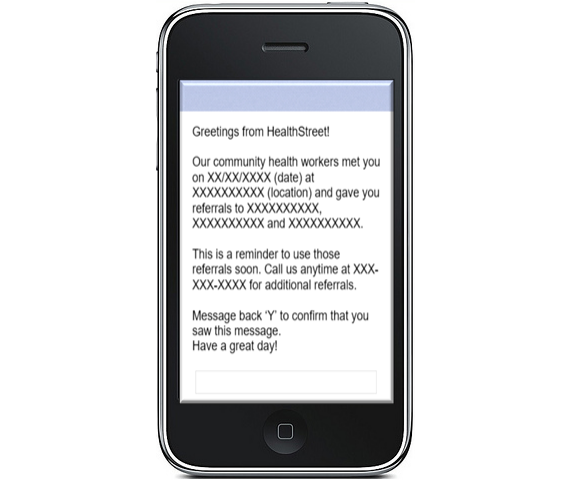
Script for text message reminder.

This text messaging software is installed for this project on a computer at HealthStreet, Gainesville. An excel spread sheet with the participant’s name, date of contact, and referral details generated by the REDCap database, is synchronized with a service that pushes out and tracks the messages. The secure encrypted database houses the participant’s name, phone number, and service referral record, while separating it by various cohorts based on the date of referral and follow-up. On the designated day for text reminders, a predesigned template message (Figure 3) reminding them of their contact with the CHWs is used by the study coordinator to enter the participant’s date, venue of contact with the CHW, and the list of referrals provided to him/her during the health intake. This message also prompts the participant to call HealthStreet in case they need more information regarding the referrals. This text messaging service is confidential and has the ability to also be embedded as a social media message, to use Quick Response codes if required, as well as track the delivery and receipt of messages through back-end analytic tools.

Satisfaction survey: a brief 15-item telephone-based survey is administered to assess the satisfaction with the process of receiving periodic reminders from HealthStreet. This survey assesses the participant’s opinions regarding the content of the text message reminders received in this study, apart from the usual 60-day follow-up questions on use of the referrals received from HealthStreet. In particular, this survey aims to gather feedback about the participant’s overall satisfaction with the reminder call and/or reminder text. The survey also assesses the participant’s perception of the reminder length, frequency, and helpfulness. Finally, this survey evaluates if the participant used their referrals because of the reminders.

### Staff Recruitment and Training

The study coordinator was trained on the scripts for reminder calls, administering the telephone-based satisfaction survey, randomization procedures, and study recruitment and intervention protocols. All CHWs and staff members were trained on administering the study informed consent and randomization letter distribution. The Qualtrics SMS software was also beta-tested using the proposed text reminder scripts.

### Data Collection

There is no risk of this study interfering with HealthStreet data, because exporting this data does not affect the value of any data in HealthStreet’s dataset. In addition to using data already collected by HealthStreet, this study collects data on the results of 15/45 day texts/calls reminders, and from the satisfaction survey. This data is kept separate from HealthStreet data, and is accessible to the Principal Investigator (PI), Co-PI, research statistician, and study coordinator.

### Data Management

The data manager at HealthStreet along with the study coordinator assists the PI in all aspects of data management. All data are stored at HealthStreet, Gainesville and available only to the PI, Co-PI, research statistician, and the study coordinator. This study follows a data management process that is similar to what is being currently followed at HealthStreet using Microsoft Access on secured local servers. Data needed for the text messaging software are generated from a report run on HealthStreet data, without any risk of interfering with HealthStreet data quality. All data is stored in locked filing cabinets or on secure computer servers with security passwords.

### Interventions

Every Monday, the research statistician generates a list of the names, participant’s unique study identification (ID) number, contact numbers, and the list of referral services of participants who need to be contacted, either for a reminder call, text message, or for 30- or 60-day follow-up. The list is handed over to the study coordinator who takes the appropriate action for each participant, after verifying this list with the intervention tracking database to ensure that all participants on the list are indeed due for their assigned intervention. The same list and procedure is used for the program algorithm that sends text messages to the participants. All participants are assigned a HealthStreet personal identifier which is used to generate an excel sheet with the participant’s date of contact, study ID, randomization number, and 15th, 30th, 45th, and 60th day due dates for intervention.

### Sample Size

The sample size for this study is 100 participants in each arm of the study. The planning compliance rate difference is 16% for a pair of treatments (22% vs 38%). The key nuisance parameter that affects planning is the probability of the second visit being compliant given the first visit is compliant.  We shall presume a strong association for an individual, half way between independence and full dependence.  Thus, for a treatment group with a compliance rate of 22%, the distribution would be 17% comply with one of two, and 13.5% comply with both.  Under independence (complete dependence), 22% (5%) would comply to both, with the average 13.5%.  For a treatment group with a compliance rate of 38%, the distribution would be 47.1% would comply with one of two, and 14.4% comply with both. With N=100 per group, and the above repeated measures structure, we have 80% power to detect a difference of 16% (22% vs 38%) (ie, personal means for two-trials of 0.44 vs. 0.76) at *P*=.017 (two-sided). Please note that we took a conservative approach using a strong within subject association. If it was weaker, power would be somewhat better.

### Eligibility

Eligibility was determined by the following criteria: all community members between 18 and 80 years of age; all who complete a HealthStreet health intake with the CHWs in Alachua and Duval counties in North Central Florida; must use the text messaging feature on their mobile phone; and have received two or more medical or social service referrals.

### Recruitment

Approximately 40 new community members are enlisted each week and 71% of them report using text messaging. We are recruiting 300 participants from Alachua and Duval counties and considered a 15% attrition rate at the 60-day follow-up. CHWs screen each participant for text capability during the health intake using the HealthStreet intake form. All eligible participants are informed about this study after the administration of the health intake. Those who express interest are given the study-specific informed consent that requests consent for receiving reminder phone calls and text messages by the CHW. All those who give consent are randomly assigned to one of three groups (n=100 each) to receive one of the interventions. The randomization letters, generated by a statistician blinded to the assignment, are sealed and all CHWs carry these letters and are trained on dispersing them. The CHW opens the letter in the presence of the participant and explains the assignment. Thereafter, the participant dates and initials the letter. The study coordinator and PI ensure appropriate implementation of the proposed recruitment and randomization procedures throughout the study period. A detailed tracking log is being maintained to track the date on which the text messages were sent and/or reminder calls were made and the participant’s response to each of these interventions. This study has been reviewed and approved by the institutional review board at University of Florida.

### Data Analysis

Data analysis will be performed using SAS 9.4. The primary outcome of this study is use of service referrals as reported at the 30- and 60-day follow-up calls. At each time point, participants will have either used no referrals, or used one or more referrals. The data will therefore be displayed in a 3 × 2 contingency table with treatments as columns and the use of referrals as rows. Chi-square tests will be used to analyze the use of referrals by treatment group.

Secondary analysis will include ordinal logistic regression with the use of referrals as the dependent variable and predictors including age, gender, race/ethnicity, access to health care, and others as dependent variables.  This will be done separately within treatment categories. Logistic regression will also be used to analyze the number of referrals used at each time point. Additionally, the satisfaction survey data will be analyzed with respect to gender, age group, education, and type of reminders received to understand the acceptability of mobile phone–based interventions among community members.

## Results

### Current Status

Currently, the study is in the participant recruitment and follow-up phase. The authors anticipate completion of recruitment, interventions, and data entry by July 2016. The analysis of all data and the preliminary results should be available by September 2016.

**Dissemination Policy**

Study results will be published in high impact, peer-reviewed scientific journals.

## Discussion

### Trial Implications

This study aims to test the feasibility of using text messages and reminder calls to improve the use of social and medical service referrals provided to community members by CHWs. A second objective is to understand community member’s satisfaction regarding receiving text messages and calls as reminders and obtain their suggestions for improving this channel of communication.

Previous data from HealthStreet has indicated “losing the referral slip” or “forgetfulness” as one of the important reasons for not using the medical and social service referrals provided by the CHWs to HealthStreet participants. These reminders will assist in removing the above mentioned barriers to utilization. Additionally, HealthStreet is currently planning to integrate reminder texts and calls to remind HealthStreet participants of their referrals, and various events organized at HealthStreet. Findings from this study will help to understand which type of reminders–TEXTONLY, CALLSONLY, or TEXT+CALLS–are most effective, useful and acceptable to the participants. The satisfaction survey administered in this study also gives us detailed information on participant’s preferred frequency of reminders. Further, this pilot study will assist in calculating the additional personnel and infrastructure including software related-cost involved in implementing a reminder protocol at HealthStreet. Most importantly, findings from this study could be applied to other community engagement initiatives around the country.

### Conclusions

The aim of this pilot study is to assess the effectiveness of adding reminders in the form of text messaging versus standard reminder calls to increase use of service referrals provided through community outreach. This pilot project would provide an excellent opportunity to test the effectiveness of mobile-based interventions on a nonclinical, community-recruited population such as those served by HealthStreet. Currently, HealthStreet does not have a formal reminder system regarding the referrals provided during the initial contact by the CHWs. Based on the findings from this study HealthStreet would develop and implement a reminder protocol for all HealthStreet members who receive social and/or medical service referrals during community outreach, thereby increasing the effectiveness of HealthStreet in the community. Additionally, the findings from this study would be also beneficial to similar community-based engagement programs nationwide.

## Appendix

Multimedia Appendix 1 Script for reminder calls.

Multimedia Appendix 2 Protocol reviewer comments.

## Abbreviations

CHWs: community health workers
CI: confidence interval
ID: identification
HIV: human immunodeficiency virus
OR: odds ratio
PI: principal investigator
SMS: short message service
