# Supplementary material for: A Research Protocol to Test the Effectiveness of Text Messaging and Reminder Calls to Increase Service Use Referrals in a Community Engagement Program
Source: JMIR Res Protoc. 2016 Jun 28;5(2):e133. doi: 10.2196/resprot.5854 (PMC4942681; doi:10.2196/resprot.5854)
Supplement: Multimedia Appendix 1 [file resprot_v5i2e133_app1.pdf]

Effectiveness of Text Messaging and Reminder Calls to Increase Use Of Service Referrals Provided Through Community Outreach

Reminder Calls

IF THE CALL GOES TO A VOICEMAIL - GO TO PART 4 OF THE MESSAGE
IF THE CALL IS ANSWERED BY A PERSON - GO TO PART 1 OF THE MESSAGE
IF THIS IS A RETURN PHONE CALL- GO TO PART 2 OF THE MESSAGE

PART 1 OF THE MESSAGE

Hello,

This call is from HealthStreet at University of Florida. We are trying to reach Ms. ………………………………….. Is this Ms. / Mr. …………………………………..?

Answer 1: Yes this is. **CONTINUE TO PART 2 OF THE MESSAGE**

Answer 2: No, I am her/ his XXXXX (anyone other than the participant).
CONTINUE TO PART 3 OF THE MESSAGE

PART 2 OF THE MESSAGE

Hi Ms./Mr.…………………………………..

I am ……………………………………………….. (name of the HealthStreet staff who is making the call) from HealthStreet. One of our community health workers, Ms./ Mr. ………………………………….. met you on ………………………………….. (date) at XXXX (location) and did a health intake. She also gave you a referral to ……………………………………………………………………. (name), ……………………………………………………………………. (name) and ………………………………………….. ……………………….. (name). This is to remind you to please go and use those referrals at your earliest convenience. Please call us back at XXX-XXX-XXXX anytime for additional services.

Thank you
And have a great day!

PART 3 OF THE MESSAGE

This is a message for Ms./Mr. …………………………………………………………………………from HealthStreet at University of Florida. Please let her know that we called and request her to call us back at XXX-XXX-XXXX at her earliest convenience.

Thank you
And have a great day!

PART 4 OF THE MESSAGE

Hello,

This call is for Ms./Mr. ………………………………………………………………………… from HealthStreet at University of Florida. Please let her know that we called her and request her to call us back at XXX-XXX-XXXX at her earliest convenience.

Thank you
And have a great day!
