# Supplementary material for: A Research Protocol to Test the Effectiveness of Text Messaging and Reminder Calls to Increase Service Use Referrals in a Community Engagement Program
Source: JMIR Res Protoc. 2016 Jun 28;5(2):e133. doi: 10.2196/resprot.5854 (PMC4942681; doi:10.2196/resprot.5854)
Supplement: Multimedia Appendix 2 [file resprot_v5i2e133_app2.pdf]

UF CRC SCIENTIFIC ADVISORY COMMITTEE SCORESHEET

Date of SAC meeting: November 20,2014

Protocol Title: Effectiveness of text messaging and reminder calls to increase use of se

Principal Investigator: Deepthi Varma, PhD

Reviewer: [redacted]

Recommended Scoring System

Reviewers should consider each of five review criteria, and the overall impact/priority of each application, on a 9-point rating scale according to the following descriptions and additional guidance:

Impact	Score	Descriptor	Additional Guidance on Strengths/Weaknesses
High	1	Exceptional	Exceptionally strong with essentially no weaknesses
	2	Outstanding	Extremely strong with negligible weaknesses
	3	Excellent	Very strong with only some minor weaknesses
Medium	4	Very Good	Strong but with numerous minor weaknesses
	5	Good	Strong but with at least one moderate weakness
	6	Satisfactory	Some strengths but also some moderate weaknesses
Low	7	Fair	Some strengths but with at least one major weakness
	8	Marginal	A few strengths and a few major weaknesses
	9	Poor	Very few strengths and numerous major weaknesses

After considering all of the review criteria, briefly assess the significant strengths and weaknesses of the application and state the likelihood of the project to exert a sustained powerful influence on the field.

Base your score on the above numbers, 1 – 9, rounding up to single decimal.

1. Significance. Does the project address an important problem or a critical barrier to progress in the field? If the aims of the project are achieved, how will scientific knowledge, technical capability, and/or clinical practice be improved? How will successful completion of the aims change the concepts, methods, technologies, treatments, services, or preventative interventions that drive this field?

3

COMMENTS

---

2. Investigator(s). Are the PD/PIs, collaborators, and other researchers well suited to the project? If Early Stage Investigators or New Investigators, or in the early stages of independent careers, do they have appropriate experience and training? If established, have they demonstrated an ongoing record of accomplishments that have advanced their field(s)? If the project is collaborative or multi-PD/PI, do the investigators have complementary and integrated expertise; are their leadership approach, governance and organizational structure appropriate for the project? Does the investigator have the time and administrative support to do this project?

1

COMMENTS

---

3. Innovation. Does the application challenge and seek to shift current research or clinical practice paradigms by utilizing novel theoretical concepts, approaches or methodologies, instrumentation, or interventions? Are the concepts, approaches or methodologies, instrumentation, or interventions novel to one field of research or novel in a broad sense? Is a refinement, improvement, or new application of theoretical concepts, approaches or methodologies, instrumentation, or interventions proposed?

2

COMMENTS

---

4. Approach. Are the overall strategy, methodology, and analyses well-reasoned and appropriate to accomplish the specific aims of the project? Are potential problems, alternative strategies, and benchmarks for success presented? If the project is in the early stages of development, will the strategy establish feasibility and will particularly risky aspects be managed?

1

Are the plans for 1) protection of human subjects from research risks, and 2) inclusion of minorities and members of both sexes/genders, as well as the inclusion of children, justified in terms of the scientific goals and research strategy proposed?

COMMENTS

---

5. Environment. Will the scientific environment in which the work will be done contribute to the probability of success? Are the institutional support, equipment and other physical and financial resources available to the investigators adequate for the project proposed? Will the project benefit from unique features of the scientific environment, subject populations, or collaborative arrangements?

1

COMMENTS

Protections for Human Subjects. For research that involves human subjects but does not involve one of the six categories of research that are exempt under 45 CFR Part 46 (as described in Human Subjects Protection and Inclusion), reviewers are asked to evaluate the justification for involvement of human subjects and the proposed protections from research risk relating to their participation according to the following five review criteria: 1) risk to subjects, 2) adequacy of protection against risks, 3) potential benefits to the subjects and others, 4) importance of the knowledge to be gained, and 5) data and safety monitoring for clinical trials. If all of the criteria are adequately addressed, and there are no concerns, write "Acceptable Risks and/or Adequate Protections.". If one or more criteria are inadequately addressed, write, "Unacceptable Risks and/or Inadequate Protections" and document the actual or potential issues that create the human subjects concern. Also, if a clinical trial is proposed, evaluate the Data and Safety Monitoring Plan. (If the plan is absent, notify the SRO immediately to determine if the application should be withdrawn.) Indicate if the plan is "Acceptable" or "Unacceptable", and, if unacceptable, explain why it is unacceptable.

Please underline one: Acceptable / Unacceptable

COMMENTS

Final Recommended Impact Score 2

UF CRC SCIENTIFIC ADVISORY COMMITTEE SCORESHEET

Date of SAC meeting: November 20, 2014

Protocol Title: Effectiveness of Text Messaging and Reminder Calls to Increase use of

Principal Investigator: Deepthi Varma, PhD

Reviewer: [redacted]

Recommended Scoring System
Reviewers should consider each of five review criteria, and the overall impact/priority of each application, on a 9-point rating scale according to the following descriptions and additional guidance:

Impact	Score	Descriptor	Additional Guidance on Strengths/Weaknesses
High	1	Exceptional	Exceptionally strong with essentially no weaknesses
	2	Outstanding	Extremely strong with negligible weaknesses
	3	Excellent	Very strong with only some minor weaknesses
Medium	4	Very Good	Strong but with numerous minor weaknesses
	5	Good	Strong but with at least one moderate weakness
	6	Satisfactory	Some strengths but also some moderate weaknesses
Low	7	Fair	Some strengths but with at least one major weakness
	8	Marginal	A few strengths and a few major weaknesses
	9	Poor	Very few strengths and numerous major weaknesses

After considering all of the review criteria, briefly assess the significant strengths and weaknesses of the application and state the likelihood of the project to exert a sustained powerful influence on the field.

<u>Base your score on the above numbers, 1 – 9, rounding up to single decimal.</u>

1. Significance. Does the project address an important problem or a critical barrier to progress in the field? If the aims of the project are achieved, how will scientific knowledge, technical capability, and/or clinical practice be improved? How will successful completion of the aims change the concepts, methods, technologies, treatments, services, or preventative interventions that drive this field?

2

COMMENTS

Interventions to improve service utilization are important. This low cost intervention could produce important increases in utilization, thereby improving participant health.

2. Investigator(s). Are the PD/PIs, collaborators, and other researchers well suited to the project? If Early Stage Investigators or New Investigators, or in the early stages of independent careers, do they have appropriate experience and training? If established, have they demonstrated an ongoing record of accomplishments that have advanced their field(s)? If the project is collaborative or multi-PD/PI, do the investigators have complementary and integrated expertise; are their leadership approach, governance and organizational structure appropriate for the project? Does the investigator have the time and administrative support to do this project?

1

COMMENTS

The PI is a junior investigator with strong trinaing and relevant experience. The mentor, Dr. Cottler, is ideally suited to assist with the project, and Dr. Hart brings valuable expertise for developing the messaging.

3. Innovation. Does the application challenge and seek to shift current research or clinical practice paradigms by utilizing novel theoretical concepts, approaches or methodologies, instrumentation, or interventions? Are the concepts, approaches or methodologies, instrumentation, or interventions novel to one field of research or novel in a broad sense? Is a refinement, improvement, or new application of theoretical concepts, approaches or methodologies, instrumentation, or interventions proposed?

4

COMMENTS

These methods have been widely employed in health care and other research settings. Their application to a community population is somewhat novel.

4. Approach. Are the overall strategy, methodology, and analyses well-reasoned and appropriate to accomplish the specific aims of the project? Are potential problems, alternative strategies, and benchmarks for success presented? If the project is in the early stages of development, will the strategy establish feasibility and will particularly risky aspects be managed?

2

Are the plans for 1) protection of human subjects from research risks, and 2) inclusion of minorities and members of both sexes/genders, as well as the inclusion of children, justified in terms of the scientific goals and research strategy proposed?

COMMENTS

The intervention seems well-conceived and the methods are sound. The exploratory analyses to identify factors predicting effectiveness of the intervention are a strength. A minor concern is that the text messaging system, telephone scripts, and satisfaction survey are yet to be developed, and the analysis plan does not address the satisfaction data. Also, it is possible that attrition will be higher than the expected 15%.

5. Environment. Will the scientific environment in which the work will be done contribute to the probability of success? Are the institutional support, equipment and other physical and financial resources available to the investigators adequate for the project proposed? Will the project benefit from unique features of the scientific environment, subject populations, or collaborative arrangements?

1

COMMENTS

HealthStreet provides the ideal environment for this project.

Protections for Human Subjects. For research that involves human subjects but does not involve one of the six categories of research that are exempt under 45 CFR Part 46 (as described in Human Subjects Protection and Inclusion), reviewers are asked to evaluate the justification for involvement of human subjects and the proposed protections from research risk relating to their participation according to the following five review criteria: 1) risk to subjects, 2) adequacy of protection against risks, 3) potential benefits to the subjects and others, 4) importance of the knowledge to be gained, and 5) data and safety monitoring for clinical trials. If all of the criteria are adequately addressed, and there are no concerns, write "Acceptable Risks and/or Adequate Protections.". If one or more criteria are inadequately addressed, write, "Unacceptable Risks and/or Inadequate Protections" and document the actual or potential issues that create the human subjects concern. Also, if a clinical trial is proposed, evaluate the Data and Safety Monitoring Plan. (If the plan is absent, notify the SRO immediately to determine if the application should be withdrawn.) Indicate if the plan is "Acceptable" or "Unacceptable", and, if unacceptable, explain why it is unacceptable.

Please underline one: Acceptable / Unacceptable

COMMENTS

There was no human subjects section in my packet. The only potential concern is how the investigators will protect against PHI disclosures if participants share their phone with other.

Final Recommended Impact Score 2
